# Measurement of Behavioral Emotion Regulation Strategies in Early Childhood: The Early Emotion Regulation Behavior Questionnaire (EERBQ)

**DOI:** 10.3390/children8090779

**Published:** 2021-09-06

**Authors:** Nicole B. Perry, Jessica M. Dollar

**Affiliations:** 1 Human Development and Family Sciences, University of Texas at Austin, Austin, TX 78712, USA; 2 Kinesiology and Psychology, University of North Carolina at Greensboro, Greensboro, NC 27412, USA; jmdollar@uncg.edu

**Keywords:** emotion regulation, behavioral regulation, strategies, early childhood, measurement

## Abstract

The Early Emotion Regulation Behavior Questionnaire (EERBQ) assesses children’s emotion regulation (ER) behavioral strategies in both positive and negative emotional contexts. Psychometric properties and factor structure were tested in a sample of caregivers across the United States (*N* = 362) with children ages 2–6 years-old (56% male; 73% White). Findings suggest that the EERBQ is psychometrically sound and correlates with other well-established measures of children’s socioemotional functioning. Previously, researchers have only been able to assess children’s emotional behavioral regulatory strategies in a laboratory setting. Thus, use of the EERBQ addresses a critical gap in the current literature by providing researchers and practitioners with an instrument to measure young children’s early emotional functioning outside of a laboratory context. This is particularly salient because early difficulty regulating emotions is often a precursor to persistent adverse developmental outcomes. Thus, the ability to easily to collect rich and predictive behavioral regulation data is imperative for early identification and treatment of youths’ emotional and behavioral problems.

## 1. Introduction

The development of emotion regulation (ER) is a critical accomplishment in early childhood given its integral role in normative and atypical development [1]. Researchers have documented the relation between ER and adjustment across both social and emotional domains [2,3], and empirically linked difficulties in ER to early indices of psychopathology including internalizing [4,5] and externalizing behavior problems [6,7]. The emphasis on ER as a predictor of behavior problems is logical given that excessive emotional reactivity, and a lack of behavioral and emotional control, are considered core symptoms for children with externalizing-type behaviors [8], and children with internalizing spectrum problems have shown difficulty regulating fear and negative arousal [9], and often use ER behavioral strategies in a hypervigilant way to suppress the expression of negative affect [10].

Consistent with many of our colleagues [11,12,13], we define ER as a set of processes that function at biological, behavioral, and social levels. Specifically, these processes capture dynamic behaviors and complex biological responses that are both automatic and effortful, as well as conscious and unconscious. They serve to modulate, maintain, inhibit, or enhance the intensity and valence of emotional experiences in an effort to accomplish an individual’s goals. Although ER processes are dynamic and function on multiple levels, significant methodological challenges often hinder our ability to investigate the development of ER as a process that incorporates all components of biology, behavior, and the environment simultaneously, often leaving scientists to focus on individual components, or the associations between these components as they relate to regulatory functioning.

In early childhood, one of the most common ways of measuring ER is through observational laboratory assessment, likely because observational indicators of emotional expression and regulation may be more informative and age appropriate during this period than during middle childhood or adolescence when regulation abilities become more internalized and display rules influence the behavioral indicators of ER [14]. Moreover, researchers can attempt to evoke specific emotions in laboratory settings and directly observe variation in children’s behavior to the same social context. Although important and informative, these observational assessments require considerable time and financial resources. They also only provide a snapshot of children’s behavior in a novel environment, and do not necessarily reflect regulatory behaviors across different social and emotional contexts, especially those in which the child is comfortable. Finally, coming to the laboratory can be taxing for caregivers in that it can require missing work, finding transportation, and organizing childcare for siblings, resulting in lower participation rates by vulnerable populations and creating greater biases in empirical work examining the early development of ER. 

Caregiver-report questionnaires can be used as an alternative to, or in combination with, observed laboratory assessments. However, existing questionnaires are limited in that they measure the extent to which children are generally well-regulated or dysregulated without providing insight into the specific regulatory behaviors children employ when emotionally aroused. Given that children’s regulatory strategies are theorized to become increasingly more sophisticated across early childhood [15], and that early developmental trajectories of independent ER strategies are often a precursor to persistent adverse developmental outcomes, the ability to easily to collect rich and predictive behavioral regulation data is imperative for early identification and treatment of youths’ emotional and behavioral problems.

Further, ER includes maintaining, enhancing, or reducing *both* positive and negative emotion in order to meet the goals of the individual and behave in a socially appropriate manner, and empirical work using caregiver-report measures provides evidence that different regulatory behaviors may emerge depending on the emotional context [16]. Yet, few existing caregiver-report questionnaires assess the regulation of specific positive and negative emotions, leaving a gap in our ability to assess regulatory behaviors that assist in modulating positive emotion. 

The Early Emotion Regulation Behavior Questionnaire (EERBQ) addresses multiple weaknesses in existing childhood ER measurement tools by using caregiver report to assess the use of specific ER strategies across everyday situations that elicit either positive (i.e., excitement, joy) or negative (i.e., anger, fear, sadness) emotion in children aged 2 to 6. We believe the EERBQ will allow for greater insight regarding the development of behavioral strategies, how behavioral strategies might differ across commonly occurring positive and negative social contexts, and the way in which distinct behavioral strategies may be associated with later adjustment. Thus, the goal of this paper is to preliminarily examine the EERBQ’s psychometric properties, factor structure, and demographic and adjustment correlates. 

### 1.1. ER Behaviors in Early Childhood

Due to relatively limited cognitive abilities in early childhood, the regulatory strategies that young children employ to manage their emotions can range from rudimentary to increasingly sophisticated and are largely behaviorally based (e.g., observable) [17]. Both rudimentary and more sophisticated behavioral strategies serve to reduce emotional arousal and intensity. However, less sophisticated regulatory behaviors require little cognitive control or effort. These strategies reduce arousal but fail to support adaptive social interaction. Thus, although the intensity of the emotion is decreased, it is done in such a way that leads to increased social difficulties. Physical and verbal venting behaviors, for example, are commonly observed when a child is angry or frustrated; physically hitting an object or person dampens emotional intensity, but these behaviors are not received well in social situations. 

Increases in brain development and cognitive functioning often forecast an increase in more sophisticated and socially appropriate behaviors, which are more likely to foster interpersonal interaction and aid children in achieving social goals [18]. Advancement in executive functioning, for instance, helps children anticipate the effectiveness of behavioral strategies and assess their own ability to handle the situation independently. Importantly, even though children become more capable of independent ER over time, ER is not always accomplished alone; children can utilize caregivers to assist in the regulation of emotion [18]. For example, when a child feels emotionally overwhelmed by a situation, they may strategize to seek help from a trusted caregiver, including physical help-seeking strategies (i.e., asking for a hug or holding a caregiver’s hand). As cognitive capacities mature, children may employ more advanced verbal help-seeking strategies such as engaging in dialogue with a caregiver (e.g., asking direct questions pertaining to the situation) that they know will help calm or relax them. From a developmental perspective, a child who initiates a conversation or contact with a caregiver during a stressful situation is quite different from a child relying on a parent to intervene and initiate the manner in which the child’s arousal is reduced. 

With maturation of attentional neural networks, children begin to effortfully redirect attention using distraction strategies, such as shifting attention to less emotionally relevant aspects of a situation or engaging in an entirely new activity [18], both of which are thought to successfully reduce negative arousal. As young children acquire greater reflective skills, they are more likely to be mindful of how they are feeling, which then may help them to strategize ways to transform a distressing emotional situation into a manageable one. For instance, as mindfulness of their own emotional experiences increases, children can brainstorm solutions to a problem, or plan a course of action, both of which may reduce the stress and arousal of a situation and/or manage consequences that arise as a result of a negative event [19]. 

Overall, there is ample evidence that young children use multiple behavioral regulation strategies to manage their emotions across various contexts. Although theoretical work posits that these strategies become increasingly sophisticated with age and increased cognitive function [20], current measurement restraints have made it challenging to repeatedly assess strategy use and document these changes within children over time, a necessary requirement for a more complete developmental profile. The EERBQ allows researchers to identify developmental shifts in ER behavioral strategies across childhood both within and across children. Moreover, this measure provides the opportunity to empirically investigate the associations between regulatory *behavioral patterns* and functioning across cognitive and social domains, both of which are important to understand how early ER development serves as a mechanism toward psychopathology and/or adaptation. 

### 1.2. The Regulation of Positive Emotion

The majority of ER research and measures focus solely on the regulation of negative emotion, but positive emotion also requires modulation and maintenance. There are situations in which excessive excitement or joy needs to be lowered or downregulated [21]. For instance, a child who becomes so excited that their behavior becomes disorganized and overwhelming to peers, may suffer negative social repercussions because their level of positive arousal and/or associated behavior is too taxing for those around them. 

There is also a considerable lack of understanding about which ER behavioral strategies children use to regulate positive emotions. It is unclear whether children use similar strategies to regulate positive and negative arousal, or if entirely different behaviors are employed. We also know very little about whether children are more or less efficient at regulating positive versus negative emotion, or the extent to which individual differences emerge across positive and negative emotional contexts. For example, it is important to know whether children find it more difficult, on average, to behaviorally manage negative arousal than positive arousal, or whether the intensity of the emotion (regardless of whether it is positive or negative) is the primary predictor of later adjustment. 

Addressing these crucial questions will greatly improve prevention and intervention efforts by highlighting that “more is not always better” in terms of positive affect, and will elucidate whether different regulatory strategies should be promoted to help children learn how to acknowledge and regulate positive and negative affect. 

### 1.3. Current Measures of ER in Early Childhood

#### 1.3.1. Caregiver-Report Questionnaires

A significant number of caregiver-report questionnaires are used in ER research including the Infant Behavior Questionnaire [22], the Toddler Behavior Questionnaire [23], the Child Behavior Questionnaire [22,24], and the Child Behavior Checklist [25]. However, scales from these measures assess specific dimensions of temperament including negative affectivity, emotional reactivity, and general self-regulation without a focus on the behavioral strategies used in the regulation of emotion during early childhood. 

The most frequently used caregiver-report measure specific to the regulation of emotion is the *Emotion Regulation Checklist* (ERC) [26], which yields Emotion Regulation and Negative Reactivity/Lability subscales. Although useful, similar to the aforementioned measures, the ERC provides a general account of children’s ER abilities and does not provide information on the regulation of specific emotions (positive and negative) or the behavioral strategies children employ to regulate emotion. Importantly, the Emotion Regulation subscale of the ERC has consistently shown lower internal reliability in early childhood samples [27,28], likely because it was originally designed for use with children in middle childhood and early adolescence.

*The Emotion Questionnaire*, put forth by Rydell and colleagues [16], is another distinct measure of ER and emotionality in children aged 5 to 8. Unlike the ERC, the *Emotion Questionnaire* does consider ER separately for anger, sadness, fear, and excitement. Again, however, this questionnaire is intended to measure the general extent to which children are emotionally reactive or able to regulate across these emotional contexts. It also does not address behavioral strategies that shed light on *how* children regulate their emotions and does not provide researchers with a tool to assess how behavioral regulation might qualitatively change over time. 

#### 1.3.2. Laboratory Assessment

To date, laboratory paradigms are the only way to assess young children’s behavioral ER strategies across multiple emotional contexts. Researchers code the behavioral strategies that children use to independently regulate their arousal including self-soothing, help-seeking or orientation toward a caregiver, disengagement of attention, avoidance, approach, and venting [29,30]. It is from these observed behaviors that the EERBQ behavioral subscales are modeled. 

In a laboratory assessment, only a snapshot of regulatory behavior in a specific and unnatural context is provided. It is often assumed that strategies employed in the laboratory are similar to those that are used in everyday settings. However, the laboratory is a novel environment that, by itself, could elicit unique behavioral responses. Moreover, researchers or practitioners often do not have the resources to bring children in to the laboratory or assessment space more than once or twice across the early childhood period, making it very challenging to track the rapid change in behavioral regulation strategies that occurs during this time. Therefore, there is limited empirical evidence with regard to strategy development over time and associations with later outcomes. 

The EERBQ was developed to assess behavioral strategies outside the laboratory by having caregivers, who see children in a variety of contexts, report on the extent to which their child uses specific behaviors across positive and negative emotionally challenging situations. Moreover, the questionnaire format allows for more frequent assessment, across both typical and vulnerable populations, so that change over time can be examined and can be used in the early identification, assessment, and prevention of children’s emotional and behavioral problems.

### 1.4. The Current Study

The goal of the current study is to examine the EERBQ’s psychometric properties and factor structure, as well as correlations with demographic variables and other measures of social and emotional adjustment. The EERBQ allows scientists and practitioners to better understand (1) the age at which specific ER strategies are most common, (2) how ER behavioral strategies may evolve over early childhood into the more sophisticated strategies we see in middle childhood and adolescence, (3) if different behavioral strategies are used when experiencing distinct emotions (i.e., fear vs. anger, excitement vs. sadness), and (4) how specific behavioral strategies, and potentially the change in these strategies over time, are associated with functioning across social, emotional, and cognitive developmental domains.

## 2. Methods

### 2.1. Participants

The sample consisted of 362 caregivers, primarily mothers (352 mothers, 8 fathers, 2 grandmothers), of typically developing 2- to 6-year-old children (mean age = 50.4 months; 24% age 2, 23% age 3, 21% age 4, 20% age 5, 12% age 6; 73% White, 2% Black, 1% Hispanic, 8% Asian, and 15% Multiracial; 56% male). Caregivers reported on children’s neurodivergent diagnoses, which were then used as an exclusion criterion (*N* = 26). Caregivers were largely from the United States (2% from New England, 8% from the Mid-Atlantic region, 44% from the Midwestern region, 32% from the Southern region, 3% from the Southwestern region, and 8% from the Western region). Three percent of the sample was not from the United States. Caregivers were on average 35.62 years-old (*SD* = 5.04; range = 20–69 years); 82% White, 2% Black, 2% Hispanic, 11% Asian, and 3% Multiracial; highly educated (2% received a high school diploma or GED, 14% attended some college, 24% earned a 4-year degree, 5% attended some graduate school, 20% earned a master’s degree, and 35% earned a doctoral or professional degree), and living in a two-parent household (96%). 

### 2.2. Procedures

#### 2.2.1. Instrument Construction

The structure of the EERBQ has been used in other well-established measures of emotional responding (i.e., *Coping with Children’s Negative Emotions Scale* [31]). The eight behavioral strategies were chosen based on conceptual definitions of ER strategies and behaviors routinely observed during laboratory assessment. To ensure the measure captured a broad range of emotionally charged contexts that children encounter regularly in their day-to-day lives, we asked parents to identify typical scenarios that elicited strong positive and negative emotional responses from their children. Upon construction, several developmental scientists, who are considered experts in emotional development, reviewed questionnaire items for face validity. 

The final version of the EERBQ presents caregivers with 12 hypothetical scenarios that represent common emotionally evocative events (all items can be found in supplemental materials); three scenarios that elicit *anger*, three scenarios that elicit *fear*, three scenarios that elicit *sadness*, and three scenarios that elicit *excitement* (full questionnaire can be found in supplemental materials). For every emotional scenario, caregivers are asked to rate the likelihood that their child would engage in each of the eight behavioral regulatory strategies using a 1 (*very unlikely*) to 7 (*very likely*) Likert scale. Thus, the questionnaire yields eight subscales that delineate qualitatively different behavioral responses to positive and negative emotions. Subscales are generated by creating a mean score of the 12 items associated with each of the behavioral regulatory strategies. 

*Distraction* strategies reflect the child’s ability to independently avert attention away from the source of distress to an activity or object less arousing (e.g., find crayons or another available toy to play with while waiting to receive an exciting gift). *Mindfulness* reflects a self-awareness of one’s own feelings (e.g., acknowledging feeling frustrated/angry when told to turn off a favorite television show). *Verbal Help-Seeking* reflects eliciting caregiver assistance in dampening emotional arousal through verbal communication such as conversation or questioning (e.g., initiating a conversation with a caregiver about what will happen, or what reward will follow, while waiting to receive a shot at the doctor’s office). *Physical Help-Seeking* reflects eliciting caregiver assistance in dampening emotional arousal through physical contact such as hugs or holding (e.g., asking to hold a caregiver’s hand while in the presence of a character or person in a mask or costume). *Avoidance* reflects withdrawing from, hiding, or avoiding the source of arousal (e.g., becoming quiet, withdrawn, or retreating to a personal space upon losing a prized possession). *Verbal Venting* reflects releasing arousal in a verbal way such as yelling, screaming, or crying (e.g., screaming in a grocery store because a caregiver will not purchase a desired snack). *Physical Venting* reflects releasing arousal in a physical way such as running, jumping, kicking, or hitting (e.g., hitting another child on the playground when they will not share ball). *Self-Soothing* reflects a physical mechanism that is independently generated to calm oneself such as sucking or rubbing (e.g., sucking a thumb or cuddling a blanket during a scary thunderstorm).

An overall Negative Emotional Reactivity subscale also is derived from an additional six items rated on a 1 (*strongly disagree*) to 7 (*strongly agree*) Likert scale at the end of the EERBQ. Example items include “my child has strong emotional reactions,” “I find it easy to get my child to calm down,” and “I anticipate my child will react poorly when something upsets him/her.”

#### 2.2.2. Data Collection

Caregivers were recruited to participate via online flyers posted on social media platforms. Participants self-selected into the study. The only criterion for participation was having a child between the ages of 2 and 6 years old. Identifying information, including parents’ names, were not collected. Participation entailed completing a 15 min online survey that consisted of the EERBQ and a demographic questionnaire, in addition to the Emotion Regulation Checklist (ERC) [26] and the Strengths and Difficulties Questionnaire (SDQ) [32] to establish construct validity. Upon completion of the survey, caregivers were given the option to enter their email into a random drawing to win 1 of 4 online gift cards. Participant emails were not linked to their data. The study was approved by the Institutional Review Board at The University of Minnesota (IRB protocol number 00009842).

### 2.3. Measures

*Emotion Regulation Checklist* (ERC) [26] assesses parents’ perceptions of their child’s ER and emotionality. The ERC is composed of 24 items rated on a four-point Likert scale that indicate the frequency of emotion-related behaviors from 1 (*never*) to 4 (*always*). The ERC yields two subscales: ER and Negativity/Lability. The ER subscale is comprised of eight items and refers to a child’s ability to modulate emotional arousal. The lability/negativity subscale is comprised of 16 items and assesses negative affect, dysregulation of emotion, and emotional intensity.

The Strength and Difficulties Questionnaire (SDQ) [32] consists of 25 items describing positive and negative attributes of children. Items are allocated to five subscales of five items each: the emotional problems subscale, the conduct problems subscale, the hyperactivity subscale, the peer problems subscale, and the prosocial behavior subscale. Each item is scored on a three-point scale with zero = “not true”, one = “somewhat true”, and two = “certainly true”. Higher scores on the prosocial behavior subscale reflect strengths, whereas higher scores on the other four subscales reflect difficulties. A total difficulties score is calculated by summing the scores on the emotional symptoms, conduct problems, hyperactivity–inattention, and peer problems subscales. 

### 2.4. Data Analysis

Descriptive statistics and internal reliabilities were calculated for each EERBQ subscale as well as for the ERC and the SDQ. Correlations were also run among all subscales of all measures. The factor structure of the EERBQ was then tested. An all-inclusive single confirmatory factor analysis (CFA) model that accounts for cross-loadings and residual covariances across factors is the ideal analytic strategy. However, in a full CFA model, 219 parameters would be estimated with a sample size of only 362 participants, resulting in a very low ratio of sample size to parameter estimates, which introduces concern regarding the stability of the estimates. Moreover, given the format and structure of the EERBQ, we would expect collinearity between constructs (i.e., behavioral strategies), which results in a poor fitting all-inclusive model. Thus, CFAs were conducted separately on the covariance matrix of the 12 raw item scores associated with each behavioral strategy. We feel this strategy is particularly useful given that researchers and practitioners are unlikely to have the sample size needed to fit an all-inclusive CFA measurement model and will instead use the data to calculate separate mean composite subscales for each behavioral strategy. Thus, it is imperative to identify the factor structure of the items included in the subscales that are used in practice. Analyses were conducted in Mplus (Version 8) [33] and full information maximum likelihood (FIML) was used to handle missing data. Less than 3% of data were missing overall. 

## 3. Results

### 3.1. Internal Consistency and Interrelations of EERBQ Behavioral Subscales

Table 1 presents the descriptive statistics and internal reliability alphas for the EERBQ, ERC, and SDQ subscales. The internal reliability for the EERBQ scales were acceptable with alphas ranging from 0.75 (negative emotional reactivity) to 0.95 (self-soothing). Reliability estimates for the well-established subscales were generally adequate, with the ERC ER subscale showing the lowest level of internal reliability (α = 0.59).

The correlations among the eight EERBQ behavioral subscales and the EERBQ emotional reactivity subscale are reported in Table 2. As expected, more sophisticated strategies (distraction, mindfulness, verbal and physical help-seeking) were correlated, and less sophisticated strategies (avoidance, physical and verbal venting) were correlated. In general, the more sophisticated EERBQ subscales were not associated with the less sophisticated EERBQ subscales. When significant negative correlations were present, they were relatively modest, providing further evidence that the behavioral strategies are distinct and qualitatively different from one another.

### 3.2. Factor Structure of EERBQ

**Figure 1:**
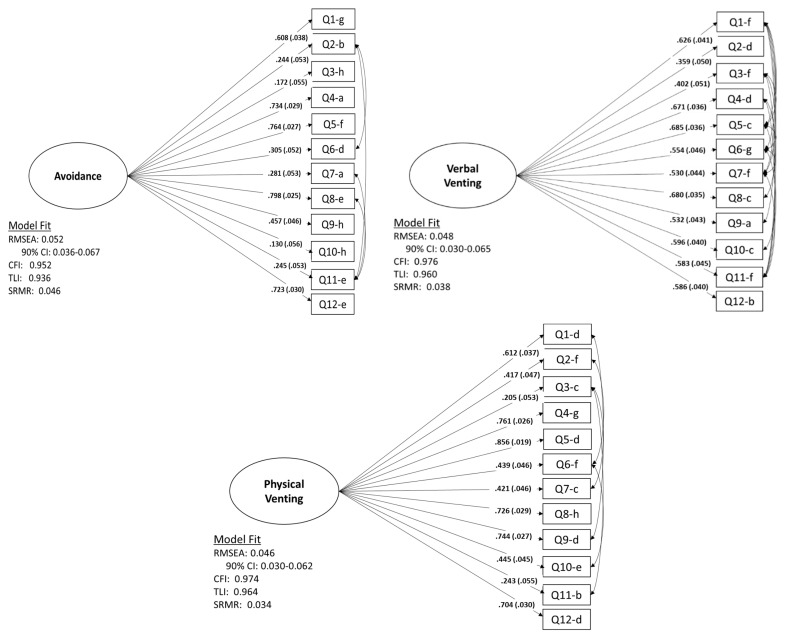
CFA measurment models with standardized estimates. Q: Question.

**Figure 2:**
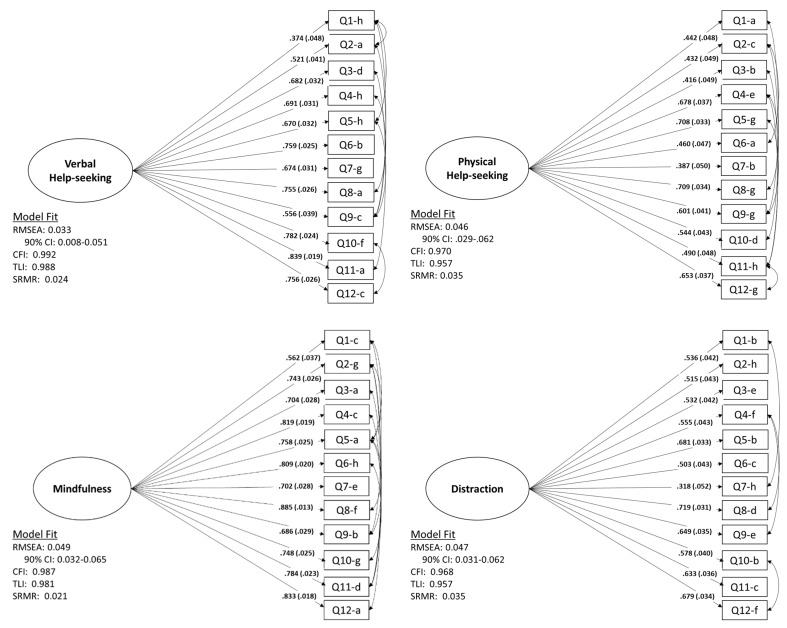
CFA Meeasurement models with standardized estimates.

In the initial CFA models, the error terms were not free to correlate. However, model fit indices suggested that fit could be improved for most factors by allowing correlation across items. The correlations across items is, in part, likely capturing methodological similarities (i.e., a similar question format) across constructs. Thus, we then ran each model allowing correlation among error terms. This resulted in excellent model fit for all behavioral strategy factors, with the exception of Self-Soothing (model fit and standardized item loadings for factors are shown in Figure 1 and Figure 2). Thus, the self-soothing factor is not presented. It should be noted that each CFA contains at least one item with lower than desired factor loadings. However, because the format of the questionnaire is such that there is a single question related to each behavioral strategy for each scenario, removing single items from the questionnaire would result in an unbalanced structure. Therefore, because the model fit was still good with these items included, we chose to retain them in the final analyses.

### 3.3. Relation of EERBQ to Parent and Child Demographics

Given the wide age range, we first considered whether child age was associated with differences in reliability statistics of the EERBQ subscales. As can be seen in Table 3, internal consistencies were comparable across ages. We then examined the role of child age in the EERBQ subscales. Child age was positively correlated with mindfulness (*r* = 0.36, *p* = 0.00), distraction (*r* = 0.11, *p* = 0.03), and verbal help-seeking (*r* = 0.41, *p* = 0.00), and negatively correlated with verbal venting (*r* = −0.25, *p* = 0.00) and physical venting (*r* = −0.19, *p* = 0.00). This indicates that, on average, older children were more likely to engage in mindfulness and verbal help-seeking, and less likely to engage in verbal and physical venting. The means and standardizations for each behavioral strategy across age (see Table 3) also support these correlations. As expected, mean distraction, mindfulness, and verbal help-seeking scores increased as children got older, while physical venting and verbal venting scores decreased as children aged. This finding provides evidence that the EERBQ is sensitive to change in children’s regulatory strategy use.

Next, we examined whether there were mean differences in EERBQ subscales when comparing boys and girls. T-tests revealed that on average, when compared to boys, caregivers reported girls to engage in more mindfulness (*t* (360) = 2.4, *p* = 0.02), distraction (*t* (360) = 2.0, *p* = 0.04), verbal *t* (360) = 2.6, *p* = 0.01) and physical (*t* (360) = 2.5, *p* = 0.01) help-seeking, and less physical venting (*t* (360) = −2.2, *p* = 0.03). In addition, girls were rated to be less emotionally reactive (*t* (360) = −2.0, *p* = 0.04) than boys. There were no significant differences in EERBQ subscales by parental education.

### 3.4. Comparison of EERBQ Subscales across Emotional Contexts

CFAs across emotional contexts would only allow for three factor loadings per behavioral strategy and we would not be able to statistically compare CFAs across emotional contexts. Thus, we decided to examine mean differences and test whether caregivers reported children to be equally likely to display each behavioral regulatory strategy across positive and negative emotional scenarios. To do this, dependent samples *t*-tests were performed and Bonferroni corrections to *p*-values were implemented. The null hypothesis of equal means across strategy use in positive and negative emotional contexts was rejected for each behavioral subscale; on average, caregivers reported a greater likelihood of their children displaying mindfulness, distraction, verbal help-seeking, and physical venting in positive emotional contexts, and avoidance, physical help-seeking, self-soothing, and verbal venting in negative emotional contexts. The paired sample statistics can be found in Table 4.

### 3.5. Relation of the EERBQ to Other Parent Indexes of Emotional Control

The correlations between the EERBQ subscales and the ERC and SDQ subscales are presented in Table 2. Overall, support was provided for construct validity and utility of the EERBQ subscales. As expected, behavioral strategies theorized to be more sophisticated in nature showed moderate to strong positive correlations with the ERC ER subscale, and moderate to strong negative correlations with the ERC Negativity/Lability subscale. The more rudimentary strategies showed moderate to strong negative correlations with the ERC ER subscale, and moderate to strong positive correlations with the ERC Negativity/Lability subscale. The more sophisticated strategies also showed moderate to strong negative correlations with the SDQ Total Difficulties subscale, and the more rudimentary behavioral strategies showed moderate to strong positive correlations with the SDQ Total Difficulties subscale.

## 4. Discussion

Results of the current study provide initial support for the internal consistency and the construct validity of the EERBQ as a measure of young children’s behavioral ER strategies, a construct that was previously only captured in laboratory settings. The individual behavioral subscales showed good internal reliability when considering all children in the sample, as well as across each age group. Thus, the EERBQ may be a useful tool to measure the use of and change in behavioral ER strategies in children ages 2–6. 

Developmental scientists know relatively little about the way in which trajectories of specific behavioral regulatory strategies predict later emotional and behavioral functioning. A transition from rudimentary to more sophisticated behavioral regulation earlier in development, for example, may be associated with increased school readiness by kindergarten and fewer behavioral problems, which is linked to social and academic success across childhood. Thus, identifying optimal developmental patterns in early childhood may help intervention and prevention efforts aimed at mitigating emotional problems. Moreover, we know little about the emergence of maladaptive regulatory behaviors prior to the onset of psychological and behavioral disorders. Therefore, measurement tools that capture specific behavioral components of early ER, and their change over time, are critically needed to further our understanding of ER as a foundational competency, and to help identify potential ER challenges before they become well-established precursors to psychopathology. 

The questionnaire format of the EERBQ is a significant strength such that it is easily administered. The broader lens offered by the EERBQ can reduce biases and increase accessibility when laboratory visits are not desired or feasible. There is also great benefit in assessing regulatory behaviors broadly, such as across settings, in addition to observing them in the laboratory. Studies including reported measures such as the EERBQ and observed assessments would allow for insight as to whether children display similar regulatory behaviors in familiar and unfamiliar settings, something that is currently unknown. Further, it would be beneficial to know whether potential differences emerge by age. For example, it is possible that younger children employ a more universal set of behavioral strategies, but as children better understand social expectations, behaviors children employ in an unfamiliar laboratory setting may become distinctly different from those that are used in familiar day-to-day scenarios. 

CFAs across behavioral subscales showed excellent model fit, with the exception of the self-soothing scale. This suggests that the individual items on the EERBQ reflect each larger behavioral strategy construct that they were designed to indicate. The lack of acceptable model fit for the self-soothing subscale may be explained by the fact that self-soothing is more prevalent at younger ages and therefore has significantly less variability. Though internal reliability remained high across ages, caution should be taken when using this subscale.

Results suggest that on average, children differ in the extent to which they use behavioral strategies in positive versus negative emotion eliciting scenarios. Specifically, we found that children were more likely to display mindfulness, distraction, verbal help-seeking, and physical venting in positive versus negative emotional contexts. These findings are notable for a variety of reasons. First, although there is a growing acceptance that “more is not always better” in terms of positive emotions, there is a dearth of research identifying *if* and *how* children regulate positive emotions. Thus, these results provide some of the first insight into the strategies that children employ in everyday situations where they are excited. 

We also found that caregivers reported a higher occurrence of physical venting in positive scenarios, which initially seems counterintuitive. Yet, it is important to consider that the EERBQ defines physical venting in an exciting scenario as behaviors that physically release positive arousal, such as jumping/bouncing, or running around. Thus, physical venting in a positive scenario is not *necessarily* maladaptive, contrary to many physical venting strategies in negative scenarios (i.e., hitting, throwing items). Moreover, physical venting in situations that elicit excitement, such as at a playground or at a birthday party, often are more acceptable environments for a child physically “vent” his/her excitement. Thus, it is less likely that caregivers intervene and teach their child to regulate this arousal. 

Noteworthy, however, is that these physical venting behaviors in other environments, such as in a school classroom, the library, or a restaurant, would be considered inappropriate and thus, less adaptive. Moreover, even in environments in which physically active and loud behaviors are acceptable, this type of behavior can become disorganized and overwhelming for those around the child, making it maladaptive and potentially leading to challenging outcomes. Indeed, in research on temperamentally exuberant children, those who are high in approach-based positive affect and activity level are often found to be at risk for developing externalizing behaviors and social difficulties [34]. In response to this research, we conducted post hoc analyses and found that children who used greater physical venting in response to excitement were rated as higher in conduct problems (*r* = 0.25, *p* < 0.01) and more hyperactive (*r* = 0.29, *p* < 0.05) by their caregivers. Thus, although concurrent associations, this provides additional support for the notion that physical venting when excited and/or showing dysregulated positive affect, not just dysregulated negative affect, may be associated with later maladjustment. Additional research is warranted to establish this association, as well as investigate if and how parents socialize the regulation of positive emotion given that growing evidence indicates its importance. 

In addition to emotional context, the age and sex of the child was associated with strategy use. Older children were more likely to use behavioral strategies that required more cognitive effort and control, while younger children were more likely to employ rudimentary strategies. This has been widely accepted throughout the developmental literature, but existing empirical evidence to support this claim has been scant to date. It is important to note, however, that the correlations between age and strategy use were not particularly strong, suggesting that regulatory behaviors are not solely dependent on age of the child. Thus, there are likely many individual and extrinsic characteristics that are associated with strategy deployment and evolution. 

Caregivers were more likely, on average, to report a higher frequency of more sophisticated strategies in girls than boys. This finding was not entirely unexpected given that caregivers have been found to accept greater emotional displays from girls, and to talk to their daughters about emotions more frequently [35]. Moreover, boys have been found to be more likely to show greater displays of anger than girls [36]. The increased emotion socialization that girls experience may provide them with greater opportunities to practice regulating emotions and employing various behavioral strategies. In turn, these opportunities may equip girls with more emotional resources when managing their arousal independently. However, more work is needed to better understand sex differences in behavioral strategy use and should be a goal of future research. 

The EERBQ subscales correlated in the expected way with other well-established measures of social and emotional functioning. The strength of these associations, especially with the ERC, was particularly promising, given that the ERC has been used as a core parent-report measure of children’s general ER abilities. Thus, EERBQ captures children’s general ability to regulate their emotions while *also* measuring the specific behavioral mechanisms that are being used to regulate both positive and negative arousal. Researchers may therefore be able to use the EERBQ as a tool that allows for a measurement of both general ER abilities and emotional reactivity, as well as more specific ER behaviors, as they relate to developmental outcomes. Significant associations in the expected direction also emerged with the SDQ subscales suggesting that specific regulatory behaviors may be distinctly associated with several aspects of psychopathology and adaptive functioning. 

### Limitations and Future Directions

Although there are significant strengths in the current study, there are noteworthy limitations. First, although the sample used in the current study is comprised of caregivers from across the United States, it lacks racial, ethnic, and socioeconomic diversity. Thus, we were not able to test measurement equivalence across varying racial, ethnic, and socioeconomic groups. Future work assessing measurement equivalence is needed to establish whether the EERBQ items tap ER behaviors similarly across racially, ethically, or socioeconomically diverse samples; doing so will ensure that meaningful inferences can be drawn from the EERBQ in diverse populations. If the EERBQ does not tap ER skills similarly across groups, its utility is considerably less, and we run the risk of introducing large amounts of unacceptable error into our models. Similarly, the sample was comprised largely of mothers. Thus, additional empirical work with fathers and other caregivers is needed to understand the inter-rater reliability of this measure and increase its broader utility. 

Given our sample size, the stability of the estimates, and collinearity across constructs, we were also not able to test a single all-inclusive CFA. Thus, we are unable to identify if there are significant cross-loadings or residual covariances across factors. However, researchers and practitioners are unlikely to be able have an appropriate sample size to conduct full measurement models and will instead compute composite measures of each behavioral strategy. Results from this study support the preliminary validity of creating those composites both through well-fitting CFAs and the associations between the behavioral composites and well-established early childhood socioemotional measures.

A third limitation is our inability to conduct a longitudinal investigation. We were unable to have participants fill out the EERBQ twice to test the level of stability in reports over time. Future work using multiple data collection time points is critical to establish the extent to which the EERBQ is able to capture change across the early childhood period. Moreover, longitudinal predictive validity is needed to assess if the associations found between the EERBQ behaviors and concurrent adjustment/maladjustment remain over time. 

It is also necessary for future work to test the associations between the EERBQ subscales and observational and physiological measures of ER to better understand how closely parents’ reports of specific strategies map on to laboratory-based paradigms and the modulation of physiological arousal. This would allow for a more comprehensive understanding of the development of ER across multiple levels of functioning. 

Finally, while the EERBQ addresses significant methodological gaps, it is also important to note that its methodological weaknesses are similar to that of observational laboratory measures. For example, it is assumed that children feel the emotion specified in the context described. However, although many children may be angered by a given context, some children instead feel sad. Relatedly, we also cannot be sure that parents are accurately interpreting their children’s emotions. Research has suggested that individual’s emotional state cannot always be inferred from their emotional expression [37]. Thus, even if a parent thinks a child would indeed feel the emotion specified in the context, it is not guaranteed to be accurate. Similar to many observational methods to assess behavioral ER strategies, the EERBQ is also unable to disentangle emotional reactivity from the regulation of emotion. Future work that can incorporate physiological and neural measures in additional to behavioral components will be necessary to gain a clearer insight into their distinction.

Overall, findings suggest that the EERBQ is psychometrically sound and provides researchers with an instrument that can readily be used to collect rich and predictive data on children’s socioemotional functioning. Use of the EERBQ can advance our current understanding of the early development of ER by providing additional insight into the prevalence of each strategy across the early childhood years, the way in which behavioral regulatory patterns evolve to master the regulation of emotion, how emotion regulation behaviors may vary across positive and emotional contexts, and how specific behavioral strategies, and potentially the change in these strategies over time, are associated with developmental outcome.

## Figures and Tables

**Table 1:** Means and Standard Deviations for Study Measures.

Measure	Mean	SD	Range	α
EERBQ				
Distraction	3.32	1.08	1.00–6.67	0.85
Mindfulness	4.69	1.48	1.00–7.00	0.94
Physical Help-Seeking	4.63	1.04	1.08–7.00	0.84
Verbal Help-Seeking	4.48	1.34	1.00–6.92	0.91
Avoidance	2.42	0.88	1.00–5.00	0.77
Physical Venting	2.62	0.86	1.00–5.75	0.84
Verbal Venting	2.83	1.12	1.00–5.92	0.85
Self-Soothing	1.80	1.19	1.00–6.50	0.95
Emotional Reactivity	3.60	0.96	1.33–6.67	0.75
ERC				
Emotion Regulation	3.40	0.31	2.25–4.00	0.59
Lability/Negativity	1.92	0.35	1.00–3.27	0.83
SDQ				
Emotional Problems	0.33	0.32	0.00–1.60	0.62
Conduct Problems	0.31	0.28	0.00–1.40	0.57
Hyperactivity	0.83	0.49	0.00–2.00	0.79
Peer Problems	0.27	0.29	0.00–1.40	0.53
Prosocial Scale	1.58	0.33	0.60–2.00	0.65
Total Difficulties	0.44	0.22	0.00–1.10	0.73

Note: *N* = 362.

**Table 2:** Correlations among Study Variables.

	1	2	3	4	5	6	7	8	9	10	11	12	13	14	15	16	17
1. Distraction	--																
2. Mindfulness	0.29 *	--															
3. Physical Help-Seeking	0.31 *	0.39 *	--														
4. Verbal Help-Seeking	0.45 *	0.74 *	0.38 *	--													
5. Avoidance	0.01	0.08	0.11 *	0.02	--												
6. Physical Venting	−0.09	−0.09	−0.08	−0.18 *	0.31 *	--											
7. Verbal Venting	−0.15 *	−0.05	−0.08	−0.22 *	0.27 *	0.75 *	--										
8. Self-Soothing	−0.05	−0.04	0.18 *	−0.04	0.34 *	0.18 *	0.14 *	--									
9. Emotional Reactivity	−0.29 *	−0.10	−0.26 *	−0.19 *	0.21 *	0.47 *	0.51 *	0.05	--								
10. Emotion Regulation (ERC)	0.19 *	0.48 *	0.31 *	0.43 *	−0.18 *	−0.26 *	−0.24 *	−0.10	−0.30 *	--							
11. Negativity/Lability (ERC)	−0.28 *	−0.05	−0.24 *	−0.17 *	0.18 *	0.52 *	0.55 *	0.08	0.67 *	−0.30 *	--						
12. Emotional Problems (SDQ)	−0.11 *	0.18 *	0.09	0.13 *	0.31 *	0.11 *	0.14 *	0.16 *	0.31 *	−0.10	0.22 *	--					
13. Conduct Problems (SDQ)	−0.26 *	−0.06	−0.28 *	−0.15 *	0.19 *	0.40 *	0.41 *	0.11 *	0.59 *	−0.31 *	0.71 *	0.18 *	--				
14. Hyperactivity (SDQ)	−0.10	0.01	−0.09	−0.10 *	0.08	0.28 *	0.32 *	0.05	0.38 *	−0.14 *	0.59 *	0.11 *	0.47 *	--			
15. Peer Problems (SDQ)	−0.06	−0.19 *	−0.14 *	−0.19 *	−0.01	0.12 *	0.12 *	0.03	0.11 *	−0.33 *	0.18 *	0.14 *	0.11 *	0.08	--		
16. Prosocial Scale (SDQ)	0.29 *	0.33 *	0.32 *	0.34 *	−0.02	−0.33 *	−0.33 *	−0.06	−0.37 *	0.50 *	−0.43 *	−0.034	−0.47 *	−0.25 *	−0.29 *	--	
17. Total Problems (SDQ)	−0.20 *	−0.01	−0.16 *	−0.12 *	0.22 *	0.36 *	0.40 *	0.13 *	0.55 *	−0.33 *	0.70 *	0.54 *	0.69 *	0.77 *	0.47 *	−0.40 *	--

Note: For readability purposes, * indicates anything where *p* < 0.05 including variables that were *p* < 0.01 and *p* < 0.001.

**Table 3:** Cronbach’s alphas, means, and standard deviations for EERBQ scales across ages.

Measure	Age 2 α (N = 85)	Mean (SD)	Age 3 α (N = 84)	Mean (SD)	Age 4 α (N = 77)	Mean (SD)	Age 5 α (N = 73)	Mean (SD)	Age 6 α (N = 43)	Mean (SD)
EERBQ										
Distraction	0.85	3.23 (1.07)	0.88	3.16 (1.14)	0.84	3.48 (1.04)	0.79	3.58 (0.93)	0.87	3.62 (1.21)
Mindfulness	0.94	3.51 (1.52)	0.94	4.85 (1.38)	0.92	5.20 (1.25)	0.89	5.14 (1.10)	0.93	5.25 (1.30)
Physical Help Seeking	0.82	4.42 (0.99)	0.84	4.77 (1.02)	0.87	4.85 (1.10)	0.81	4.42 (0.98)	0.85	4.77 (1.05)
Verbal Help-Seeking	0.93	3.38 (1.48)	0.89	4.53 (1.20)	0.86	4.96 (1.03)	0.84	4.88 (0.94)	0.89	5.08 (1.10)
Avoidance	0.76	2.34 (0.79)	0.80	2.44 (0.92)	0.77	2.32 (0.86)	0.77	2.56 (0.89)	0.80	2.49 (0.95)
Physical Venting	0.84	2.86 (0.95)	0.80	2.68 (0.78)	0.85	2.56 (0.82)	0.85	2.64 (0.89)	0.77	2.24 (0.95)
Verbal Venting	0.85	3.20 (1.16)	0.84	3.01 (1.09)	0.87	2.69 (1.13)	0.84	2.66 (1.04)	0.82	2.28 (0.96)
Self-Soothing	0.95	1.93 (1.27)	0.95	1.91 (1.32)	0.94	1.73 (1.11)	0.94	1.78 (1.11)	0.96	1.54 (1.02)
Emotional Reactivity	0.69	3.54 (0.85)	0.70	3.64 (0.85)	0.71	3.50 (0.95)	0.84	3.86 (1.16)	0.76	3.42 (1.02)

**Table 4:** Paired Sample Statistics across Positive and Negative Emotional Contexts.

Paired Samples	Mean (SD)	*r*	Mean Change (SD)	t (df = 361)
Mindfulness in Positive	4.95 (1.60)	0.80 **	0.35 (1.00)	6.74 **
Mindfulness in Negative	4.60 (1.52)			
Avoidance in Positive	1.29 (0.56)	0.38 **	−1.49 (1.02)	−27.94 **
Avoidance in Negative	2.79 (1.09)		.	
Distraction in Positive	3.58 (1.26)	0.65 **	0.35 (1.01)	6.49 **
Distraction in Negative	3.23 (1.13)			
Verbal Help-Seeking Positive	5.03 (1.61)	0.79 **	0.73 (0.99)	14.08 **
Verbal Help-Seeking Negative	4.30 (1.33)			
Physical Help-Seeking Positive	4.26 (1.28)	0.55 **	−0.49 (1.15)	−8.10 **
Physical Help-Seeking Negative	4.76 (1.10)			
Self-Soothing Positive	1.38 (0.83)	0.79 **	−0.57 (0.88)	−12.38 **
Self-Soothing Negative	1.95 (1.36)			
Verbal Venting Positive	2.79 (1.54)	0.52 **	−0.05 (1.37)	−0.757 **
Verbal Venting Negative	2.84 (1.17)			
Physical Venting Positive	4.72 (1.09)	0.36 **	2.80 (1.17)	45.61 **
Physical Venting Negative	1.91 (0.96)			

Note: ** = *p* < 0.001.

## Data Availability

The data presented in this study are available on request from the corresponding author.

## Supplementary Materials

The following are available online at https://www.mdpi.com/article/10.3390/children8090779/s1, File S1: EERBQ Questionnaire.
