# Supplementary material for: Measurement of Behavioral Emotion Regulation Strategies in Early Childhood: The Early Emotion Regulation Behavior Questionnaire (EERBQ)
Source: Children (Basel). 2021 Sep 6;8(9):779. doi: 10.3390/children8090779 (PMC8467461; doi:10.3390/children8090779)
Supplement: Supplementary file 1 [file children-08-00779-s001.zip › children-1349643-supplementary.pdf]

EERBQ Questionnaire

How My Child Handles Emotions

Please indicate on a scale from 1 (very unlikely) to 7 (very likely) the likelihood that your child would respond in the ways listed for each scenario. Please read each scenario and item carefully as sometimes children behave differently in different situations. Please respond to every item (a through h). If your child does not engage the behavior(s) listed, then respond very unlikely (1). Some items list multiple potential behaviors. For example, one item says, "suck his/her thumb or cuddle his/her blanket." If your child only does one of the behaviors listed for that item, use the item scale to rate the likelihood of that behavior. If there is a scenario that your child would likely not encounter, try to think of a similar scenario your child would encounter that elicits the same emotion and answer the items based on that scenario.

1	2	3	4	5	6	7
**Very Unlikely**			**Somewhat Likely**			**Very Likely**

1. If my child is angry because s/he wants to continue screen time (e.g., T.V., tablet, phone), but it is time to turn it off, s/he would:

	1	2	3	4	5	6	7
a. initiate physical contact from me (e.g., hug, kiss, snuggle)	O	O	O	O	O	O	O
b. find another activity or toy to play with on their own	O	O	O	O	O	O	O
c. say how frustrated or angry s/he is currently feeling	O	O	O	O	O	O	O
d. hit (a person or object), throw something (e.g., remote or pillow), or throw her/himself on the floor	O	O	O	O	O	O	O
e. suck his/her thumb, pick at his/her nails or clothes, twirl his/her hair, or cuddle his/her blanket or stuffed animal	O	O	O	O	O	O	O
f. scream or yell	O	O	O	O	O	O	O
g. run out of the room, hide in his/her room or personal space, or become quiet and withdrawn	O	O	O	O	O	O	O
h. initiate a conversation with me that does not include arguing, possibly about what he/she could do instead, or what s/he was watching/doing on the screen	O	O	O	O	O	O	O

2. If my child is scared while waiting in the doctor's office, s/he would:

	1	2	3	4	5	6	7
a. initiate a conversation with me, possibly about what we will do when we leave, or about a treat s/he will receive afterward	O	O	O	O	O	O	O
b. ask to leave, hide behind a caregiver, cover his/her eyes, or become quiet and withdrawn	O	O	O	O	O	O	O
c. ask me to hold his/her hand, sit on my lap, or initiate other physical contact	O	O	O	O	O	O	O
d. scream or yell	O	O	O	O	O	O	O
e. suck his/her thumb, pick at his/her nails or clothes, twirl his/her hair, or cuddle his/her blanket or stuffed animal	O	O	O	O	O	O	O
f. run around the room, hit (a person or object), or kick his/her legs	O	O	O	O	O	O	O
g. say how scared s/he is currently feeling	O	O	O	O	O	O	O
h. find crayons or another available toy to play with while s/he waits	O	O	O	O	O	O	O

3. If my child is excited because friends and family are arriving with gifts for his/her birthday party, s/he would:

	1	2	3	4	5	6	7
a. say how excited s/he is currently feeling	O	O	O	O	O	O	O
b. initiate physical contact from me (e.g., hug, hold on to a leg)	O	O	O	O	O	O	O
c. bounce or run around the room	O	O	O	O	O	O	O
d. initiate conversation with me, possibly about how long it will take people to arrive, or what might be inside the gifts	O	O	O	O	O	O	O
e. find another activity to engage in (e.g., play with a toy, sing/hum a song, create a dance) while s/he waits for everyone to arrive	O	O	O	O	O	O	O
f. scream or yell in excitement	O	O	O	O	O	O	O
g. suck his/her thumb, pick at his/her nails or clothes, or twirl his/her hair	O	O	O	O	O	O	O
h. run out of the room, go to his/her room or personal space, or become quiet and withdrawn	O	O	O	O	O	O	O

4. If my child is sad because s/he accidently broke his/her new toy, s/he would:

	1	2	3	4	5	6	7
a. run out of the room, go to his/her room or personal space, or become quiet and withdrawn	O	O	O	O	O	O	O
b. suck his/her thumb, pick at his/her nails or clothes, twirl his/her hair, or cuddle his/her blanket or stuffed animal	O	O	O	O	O	O	O
c. say how sad s/he is currently feeling	O	O	O	O	O	O	O
d. scream or yell	O	O	O	O	O	O	O
e. initiate physical contact from me (e.g., hug, kiss, snuggle)	O	O	O	O	O	O	O
f. find a different toy to play with, or engage in a new activity on their own	O	O	O	O	O	O	O
g. kick, hit (a person or object), or throw his/herself on the floor	O	O	O	O	O	O	O
h. initiate conversation with me, possibly about how to get a new toy, or what s/he will do now that the toy is broken	O	O	O	O	O	O	O

5. If my child becomes angry because s/he is sick/hurt and can't go outside to play with his/her siblings or friends s/he would:

	1	2	3	4	5	6	7
a. say how frustrated or angry s/he is currently feeling	O	O	O	O	O	O	O
b. search out a fun activity or toy s/he can play with inside instead	O	O	O	O	O	O	O
c. scream or yell because s/he cannot go outside	O	O	O	O	O	O	O
d. hit (a person or object), kick, or throw her/himself on the floor	O	O	O	O	O	O	O
e. suck his/her thumb, pick at his/her nails or clothes, twirl his/her hair, or cuddle his/her blanket or stuffed animal	O	O	O	O	O	O	O
f. run out of the room, go to his/her room or personal space, or become quiet and withdrawn	O	O	O	O	O	O	O
g. initiate physical contact from me (e.g., hug, kiss, snuggle)	O	O	O	O	O	O	O
h. initiate conversation with me that does not include arguing, possibly about what else s/he could do, or talk with me about how being sick is not any fun	O	O	O	O	O	O	O

6. If my child is at an event and is scared of a character (e.g., clown) or person in a mask or costume, s/he would:

	1	2	3	4	5	6	7
a. initiate physical contact from me (e.g., hug, kiss, hold hand, snuggle)	O	O	O	O	O	O	O
b. initiate conversation with me, possibly about how scary the person is	O	O	O	O	O	O	O
c. walk away and find something or someone to play with to distract him/herself from the character/person in costume	O	O	O	O	O	O	O
d. run out of the room, hide behind a caregiver, cover his/her eyes, or become quiet and withdrawn	O	O	O	O	O	O	O
e. suck his/her thumb, pick at his/her nails or clothes, twirl his/her hair, or cuddle his/her blanket or stuffed animal	O	O	O	O	O	O	O
f. kick, hit (a person or object), or flail arms or legs	O	O	O	O	O	O	O
g. scream or yell	O	O	O	O	O	O	O
h. say how scared s/he is currently feeling	O	O	O	O	O	O	O

7. If my child is excited during library story time but s/he needs to remain quiet, s/he would:

	1	2	3	4	5	6	7
a. run out of the room, hide behind a caregiver, or become quiet and withdrawn	O	O	O	O	O	O	O
b. come sit next to me during the story	O	O	O	O	O	O	O
c. run around the room, bounce up and down, or physically interact with another child (push, poke, shove)	O	O	O	O	O	O	O
d. suck his/her thumb, pick at his/her nails or clothes, twirl his/her hair, or cuddle his/her blanket or stuffed animal	O	O	O	O	O	O	O
e. say how excited s/he is currently feeling	O	O	O	O	O	O	O
f. scream, or yell in excitement	O	O	O	O	O	O	O
g. initiate conversation with me, possibly about the story or what is going on in the room	O	O	O	O	O	O	O
h. distract themselves with something nearby (e.g., play with his/her shoe laces or draw pictures with them)	O	O	O	O	O	O	O

8. If my child is sad because s/he lost a prized possession (e.g., a favorite stuffed animal or blanket) s/he would:

	1	2	3	4	5	6	7
a. initiate conversation with me, possibly about where it could be or what might have happened to it	O	O	O	O	O	O	O
b. suck his/her thumb, pick at his/her nails or clothes, twirl his/her hair, or cuddle his/her blanket or stuffed animal	O	O	O	O	O	O	O
c. scream or yell	O	O	O	O	O	O	O
d. find an activity to take their mind off it, such as going outside, coloring, or playing with toys	O	O	O	O	O	O	O
e. run out of the room, go to his/her room or personal space, or become quiet and withdrawn	O	O	O	O	O	O	O
f. say how sad s/he is currently feeling	O	O	O	O	O	O	O
g. initiate physical contact from me (e.g., hug, kiss, snuggle)	O	O	O	O	O	O	O
h. kick, hit (a person or object), or throw his/herself on the floor	O	O	O	O	O	O	O

9. If my child is angry because s/he wants a toy/snack at the store and I will not buy it, s/he would:

	1	2	3	4	5	6	7
a. scream or yell as a result of not getting the toy/snack	O	O	O	O	O	O	O
b. say how angry/frustrated s/he is currently feeling	O	O	O	O	O	O	O
c. initiate conversation with me that does not include arguing, possibly about where we will go after, or something happening in the store	O	O	O	O	O	O	O
d. hit (a person or object) or throw something, or throw her/himself on the floor	O	O	O	O	O	O	O
e. find something else to do (e.g., push the cart, help put things in the cart, make up a song) that s/he finds enjoyable	O	O	O	O	O	O	O
f. suck his/her thumb, pick at his/her nails or clothes, twirl his/her hair, or cuddle his/her blanket or stuffed animal	O	O	O	O	O	O	O
g. initiate physical contact from me (e.g., hug, kiss, snuggle)	O	O	O	O	O	O	O
h. ask to leave or become quiet and withdrawn	O	O	O	O	O	O	O

10. If my child is scared because of a loud thunderstorm, s/he would:

	1	2	3	4	5	6	7
a. run out of the room, hide behind a caregiver, cover his/her eyes, or become quiet and withdrawn	O	O	O	O	O	O	O
b. find an activity or toy to play with to keep his/her mind from the storm	O	O	O	O	O	O	O
c. scream or yell	O	O	O	O	O	O	O
d. initiate physical contact from me (e.g., hug, kiss, hold hand, snuggle)	O	O	O	O	O	O	O
e. kick, hit (person or object), or flail arms and legs	O	O	O	O	O	O	O
f. initiate conversation with me, possibly about thunderstorms or where they come from	O	O	O	O	O	O	O
g. say how scared s/he is currently feeling	O	O	O	O	O	O	O
h. suck his/her thumb, pick at his/her nails or clothes, twirl his/her hair, or cuddle his/her blanket or stuffed animal	O	O	O	O	O	O	O

11. If my child is excited to play at a park, but he/she has to wait for his/her friend to arrive, s/he would:

	1	2	3	4	5	6	7
a. initiate a conversation with me, possibly about where the friends are or how long they might be	O	O	O	O	O	O	O
b. jump or run around until his/her friends arrive	O	O	O	O	O	O	O
c. find another activity to engage in (e.g., play with a toy, sing/hum a song, create a dance) while s/he waits for friends	O	O	O	O	O	O	O
d. say how excited s/he is currently feeling	O	O	O	O	O	O	O
e. hide behind a caregiver or become quiet and withdrawn	O	O	O	O	O	O	O
f. scream or yell over her excitement	O	O	O	O	O	O	O
g. suck his/her thumb, pick at his/her nails or clothes, twirl his/her hair, or cuddle his/her blanket or stuffed animal	O	O	O	O	O	O	O
h. seek physical contact from me (e.g., hug, hold on to a leg)	O	O	O	O	O	O	O

12. If my child is sad because a favorite family member or friend must leave to go home, s/he would:

	1	2	3	4	5	6	7
a. say how sad s/he is currently feeling	O	O	O	O	O	O	O
b. scream or yell	O	O	O	O	O	O	O
c. initiate conversation with me, possibly about where they are going or when they might return	O	O	O	O	O	O	O
d. kick, hit (person or object), or throw his/herself on the floor	O	O	O	O	O	O	O
e. run out of the room, go to his/her room or personal space, or become quiet and withdrawn	O	O	O	O	O	O	O
f. find an activity to take their mind off it, such coloring or playing with toys	O	O	O	O	O	O	O
g. initiate physical contact from me (e.g., hug, kiss, hold hand, snuggle)	O	O	O	O	O	O	O
h. suck his/her thumb, pick at his/her nails or clothes, twirl his/her hair, or cuddle his/her blanket or stuffed animal	O	O	O	O	O	O	O

For the next set of questions, use the following scale

1	2	3	4	5	6	7
**Strongly Disagree**			**Neutral**			**Strongly Agree**

	1	2	3	4	5	6	7
**1. My child has strong emotional reactions**	O	O	O	O	O	O	O
**2. I find it easy to get my child to calm down**	O	O	O	O	O	O	O
**3. My child seems more emotional than other children**	O	O	O	O	O	O	O
**4. My child is quickly comforted by me**	O	O	O	O	O	O	O
**5. I anticipate my child will react poorly when something upsets him/her**	O	O	O	O	O	O	O
**6. There are specific strategies I know will work to help my child relax**	O	O	O	O	O	O	O

Scoring

Emotional reactivity – 1, 2(reverse coded), 3, 4(reverse coded), 5, 6(reverse coded)
Mindfulness – 1c, 2g, 3a, 4c, 5a, 6h, 7e, 8f, 9b, 10g, 11d, 12a
Avoidance - 1g, 2b, 3h, 4a, 5f, 6d, 7a, 8e, 9h, 10h, 11e, 12e
Distraction - 1b, 2h, 3e, 4f, 5b, 6c, 7h, 8d, 9e, 10b, 11c, 12f
Verbal help-seeking - 1h, 2a, 3d, 4h, 5h, 6b, 7g, 8a, 9c, 10f, 11a, 12c
Physical help-seeking - 1a, 2c, 3b, 4e, 5g, 6a, 7b, 8g, 9g, 10d, 11h, 12g
Self-soothing - 1e, 2e, 3g, 4b, 5e, 6e, 7d, 8b, 9f, 10h,11g, 12h
Verbal venting - 1f, 2d, 3f, 4d, 5c, 6g, 7f, 8c, 9a, 10c, 11f, 12b
Physical venting - 1d, 2f, 3c, 4g, 5d, 6f, 7c, 8h, 9d, 10e, 11b, 12d
